# Cytotoxic and apoptosis-inducing effects of wildtype and mutated *Hydra* actinoporin-like toxin 1 (HALT-1) on various cancer cell lines

**DOI:** 10.7717/peerj.6639

**Published:** 2019-05-02

**Authors:** Teng Jia Ng, Michelle Yee Mun Teo, Dek Shen Liew, Paul Etim Effiong, Jung Shan Hwang, Crystale Siew Ying Lim, Lionel L.A. In

**Affiliations:** 1 Department of Biotechnology, Faculty of Applied Sciences, UCSI university, Cheras, Wilayah Persekutuan Kuala Lumpur, Malaysia; 2 Department of Medical Sciences, School of Healthcare and Medical Sciences, Sunway University, Subang Jaya, Selangor, Malaysia

**Keywords:** Cytotoxicity, Immunotoxin, Apoptosis, Pore-forming toxins, *Hydra* actinoporin, Cancer

## Abstract

**Background:**

*Hydra* actinoporin like toxin -1 (HALT-1), is a small 18.5 kDa pore forming toxin derived from *Hydra magnipapillata* which has been shown to elicit strong haemolytic and cytolytic activity when in contact with cell membranes. Due to its cytotoxic potency, HALT-1 was further investigated for its potential as a toxin moiety candidate in immunotoxin developmental efforts, ideally as a form of targeted therapy against cancer.

**Methods:**

In this study, wtHALT-1 (wild type) and its Y110A mutated binding domain counterpart (mHALT-1) were produced and evaluated for their cytotoxic and apoptotic effects on various cancer cell lines. A total of seven different tumour and non-tumour cell lines including HeLa, HepG2, SW-620, MCF-7, CCD841CoN, NHDF and HCT116 were used. Immunofluorescence assays were used to observe membrane binding and localization changes between both HALT-1 recombinant proteins based on 6xHis-tag detection.

**Result:**

Based on MTT data, mHALT-1 demonstrated a significant reduction of 82% ±  12.21% in cytotoxic activity across all cell lines after the membrane recognition domain had been mutated in comparison to the wtHALT-1. Annexin V FITC/PI assay data also indicated that HeLa, HepG2 and MCF-7 demonstrated an apoptosis-mediated cell death after being treated with wtHALT-1. Additionally, a notable difference between wtHALT-1 and mHALT-1 binding affinity was clearly observed where emission of green fluorescence along the cell membrane was observed only in wtHALT-1 treated cells.

**Discussion:**

These results suggest that mHALT-1 (Y110A) can be potentially developed as a toxin-moiety candidate for the development of future immunotoxins against various human cell-based diseases.

## Introduction

Most protein-based toxins have the potential to be developed as a toxin moiety in immunotoxin-based drugs (Aruna, 2006; Becker & Benhar, 2012; Mazor et al., 2016; Mazor, King & Oastan, 2018; Schmohl et al., 2018; Shan, Liu & Wang, 2013; Mathew & Verma, 2009). These toxin moieties generally need to internalize and translocate to the cytosol in order to achieve its cytotoxic effects, most of which function by enzymatically inhibiting protein synthesis (Tejuca et al., 2009; Virgilio et al., 2010). However, the size of toxins is generally a major hurdle, which leads to poor tissue penetration. To overcome this problem, another class of relatively small sized toxins has been explored. Cnidarian actinoporin is a potential toxin moiety candidate for immunotoxins owing to its relatively small size (18.5–20 kDa), thus allowing increased penetration and lower immunogenicity (Tejuca et al., 2009; Mariottini & Pane, 2014). Additionally, extreme stability towards proteolytic degradation also makes them good toxin conjugate candidates (Tejuca, Anderluh & Serra, 2009).

Actinoporins depend on the recognition of sphingomyelin (SM) in the cell membrane in order to lyse and permeabilize specific cell types (Schön et al., 2008). It is capable of forming pores that disrupt ion gradients, which can cause osmotic swelling, leading to cell death. Few studies have proven that the Equinatoxin II (EqtII) toxin derived from sea anemone *Actinia equina* showed significant toxicity against Erlich ascites tumours, L1210 leukemia cells and diploid lung fibroblast of the Chinese hamster. It is a potent inhibitor of papain-like cysteine proteinase and aspartic proteinase cathepsin D, which is normally found in breast cancer and nerve-related diseases (Jouiaei et al., 2015). Another study also reported anti-butyrylcholinestrasic activity from toxins derived from the Mediterranean jellyfish *Pelagia noctiluca,* which is useful in treating Alzheimer’s disease and senile dementia (Jouiaei et al., 2015). The first use of actinoporins as part of immunotoxins to kill cancerous cells involves the hemolytic fraction from the sea anemone *Stichodactyla helianthus* fused to mAbs to target carcinoembryonic antigens (CEA) (Avila, De Acosta & Lage, 1988). Unfortunately, results showed ineffective killing towards targeted cells due to excessive binding of free antibodies (Tejuca, Anderluh & Serra, 2009). While actinoporins have shown encouraging results in recent studies, its non-specific binding capability remains a major challenge when it comes to targeted therapeutic applications.

Multiple steps are involved in the mechanism of pore formation by actinoporins. The process begins when actinoporins attaches itself to a sphingomyelin associated membrane where the phosphocholine (POC) binding site plays a critical role. Then, the N-terminal translocates to the lipid-water interface where it undergoes conformational changes, detaches from the protein and inserts into the lipid membrane. Subsequently, 3 to 4 actinoporins oligomerize within the plasma membrane and a functional pore is then created by chemical cross linking of the N-terminal α-helix (Liew et al., 2015; Gutiérrez-Aguirre et al., 2004; Cosentino, Ros & García-Sáez, 2016; Rojko et al., 2015; Hasegawa et al., 2016; Subburaj et al., 2014). A recent study showed the insertion of negative charged amino acids at the N-terminal region of *Hydra* actinoporin-like toxin-1 (HALT-1) strongly reduces the cytolytic activity of the toxin, presumably due to abrogation of its binding properties (Liew et al., 2015).

Venom from *Hydra magnipapillata’s* nematocysts has strong hemolytic and phospholipase properties, which often cause long lasting paralysis and even death (Weber, Klug & Tardent, 1987). This venom is similar to venom from jellyfish, bees, lizards, scorpions and snakes (Sher et al., 2005) with over 55- toxin related sequences including neurotoxins, cytolysins (actinoporins), toxic phopholipases and peptidases with molecular weights of 25 kDa–100 kDa (Balasubramanian et al., 2012) being discovered to date. Among these toxins lies HALT, which is an α-pore-forming toxin (α-PFT) identified from the genome of *Hydra magnipapillata* (Glasser et al., 2014). HALT-1 (20.8 kDa), which is localized in stenoteles has been shown to exhibit strong haemolytic and cytolytic activity towards hematologic cells and HeLa cells (Glasser et al., 2014; Liew et al., 2015). It binds on the cell membrane and lyses the targeted cell. The conserved cluster of aromatic amino acid and an amphiphilic N-terminus in HALT-1 was reported to be important in membrane binding and pore formation (Liew et al., 2015). Therefore, in order to further analyze the hemolytic activity of HALT-1 and its potential as a toxin moiety candidate in future immunotoxins, cytotoxic assays and immunofluorescence binding localization assays were used in this study.

## Material and Methods

### Production, isolation and purification of recombinant wtHALT-1 and mHALT-1

*Hydra*’s wtHALT-1 and mHALT-1 gene sequences were previously cloned into pET28a vector and transformed into *E. coli* BL21 (DE3) for expression. Site-directed mutagenesis of wtHALT-1 was performed where tyrosine was substituted at position 110 to alanine (Liew et al., 2015). Both wtHALT-1 and mHALT-1 were isolated and cultured in LB broth with 50 mg/mL kanamycin at 37 °C. Subsequently, 1.0 mM of IPTG was used as an inducer for protein expression at 0.5–0.6 OD_600_. Pellets were harvested by centrifugation at 10,000 rpm and sonicated with 0.5 M phenylmethanesulfonyl fluoride (PMSF) as a protease inhibitor. Sonication was done with 130 watt, 20 kHz, at 10 s pulse on, followed by 20 s pulse off. Both recombinant His-tagged wtHALT-1 and mHALT-1 were separated from insoluble fractions by centrifugation for 10 mins at 10,000 rpm, 4 °C. Expressed recombinant proteins were then purified using His-spin trap kit (GE Healthcare, US). Binding buffer with 20 mM, 50 mM and 100 mM of imidazole were used to wash purified recombinant wtHALT-1 and mHALT-1, and eluted out with 500 mM imidazole. Subsequently, SDS-PAGE with 12% (w/v) separating gel and 5% (w/v) stacking gel were used to separate purified proteins. Pure fractions of recombinant wtHALT-1 and mHALT-1 were concentrated and desalted with PBS using Amicon^®^ Ultra-2ml centrifugal filter (Merck, Germany) at 8,000 rpm, 4 °C for 15 mins. Protein quantification was performed using Quick Start™ Bradford Assay (Bio-Rad, USA) at 595 nm.

### Cell culture

Seven different cell lines, HeLa (*ATCC*^®^ CCL-2™), HepG2 (*ATCC*^®^ HB-8065™), MCF-7 (*ATCC*^®^ HTB-22™), SW-620 (*ATCC*^®^ CCL-227™), HCT116 (*ATCC*^®^ CCL-247™), CCD 841 CoN (*ATCC*^®^ CRL-1790™) and normal human dermal fibroblast (NHDF) (*ATCC*^®^ PCS-201-012™) were used in this study. Each cell line was cultured in CO_2_ incubator at 37 °C and 5% CO_2_. Cells were cultured in DMEM supplemented with 10% fetal bovine serum (FBS), 60 mg/mL of penicillin and 100 mg/mL of streptomycin.

### Cytotoxicity assay

MTT 3-(4,5-dimethylthiazol-2-yl)-2,5-diphenyl tetrazolium bromide assay was performed to evaluate the cytotoxic effects of wtHALT-1 and mHALT-1 on different cell lines. Seven different cell lines were treated with wtHALT-1, mHALT-1, and camptothecin (1–5 µM) at different concentrations. Two controls comprising a negative control (medium only) and a positive control (camptothecin) were included in each set of assay. Cells (1 × 10^4^ cells/mL) were incubated for 24 h after the addition of wtHALT-1 and mHALT-1 at a concentration of 0.20 to 1.70 µM. Briefly, 50 µL of 10 mg/mL MTT was added to each well and incubated for 4 h at 37 °C with 5% CO_2._ The supernatant was then removed and formazan violet crystals were dissolved by adding 200 µL of DMSO. Absorbance was measured at 570 nm using FLUOstar Omega microplate reader (BMG Labtech, Germany) and the percentage of cell viability was calculated. Each data point represents the average viability obtained from triplicate experiments. IC_50_ values were calculated using logarithmic regression analysis in Microsoft Excel.

### Annexin V FITC/ PI apoptosis detection assay

Annexin V FITC/ PI apoptosis detection assay kit (Invitrogen, USA) was used to determine the percentage of cells within a population that are actively undergoing apoptosis. In brief, cells (1 × 10^6^ cells/mL) were harvested after treatment with wtHALT-1 and camptothecin for 24 h using IC_50._ Treatment with mHALT-1 was also carried out using the same test concentrations as wtHALT-1. Apoptosis was detected by initially staining the cells with 0.1% (v/v) Annexin V and 100 µg/mL of propidium iodide solution followed by flow cytometry analysis. Stained cell suspensions were incubated in the dark for 15 min at room temperature, 400 µL of 1×  binding buffer was added to each tube and analyzed using NovoCyte ™ 2060 flow cytometer (ACEA Biosciences, USA).

### Immunofluorescence staining assay

Immunofluorescence (IF) staining assay was done to visualize the cellular localization of wtHALT-1 and mHALT-1 binding. Indirect IF assay was performed using primary mouse anti-His tag antibody (ABM, Canada) and Alexa Fluor 488 goat anti-mouse IgG (H+L) secondary antibody (ThermoFisher, USA). HepG2, HeLa, MCF-7 and SW-620 cell lines were harvested after treatment with wtHALT-1, mHALT-1 and camptothecin for 24 h. Cells (1 × 10^6^ cells/mL) were washed with PBS 3 times after treatment and fixed with 4% paraformaldehyde followed by 3 more washes with PBST. Permeabilization was performed by incubating the cells with 0.1% triton X-100 for 10 min. Cells were then washed 3 times with PBS and blocked with 2% BSA for 45 mins to minimize unspecific binding. Cells were then incubated with anti-His tag primary antibody for 1 h to mark histidine-tagged proteins and washed with PBST 3 times, 5 mins each. Alexa Fluor 488 goat anti-mouse IgG (H+L) secondary antibody was then added and incubated for 1 h in the dark. Cells were then washed 3 times with PBS and stained with Hoechst 33342 solution for 10 mins as a nuclear counter stain. All images were captured and analyzed using a fluorescence microscope Axio Vert A1 (Carl Zeiss, Germany) under 630×  magnification. Fluorescence quantification was calculated using Zen software based on captured triplicate images.

### Statistical analysis

All statistical analysis was completed using Excel. Data were expressed as mean ± S.D. The statistical significance analysis was done using Student’s two tailed *t*-test, and data significance levels are shown as *p  < 0.05, **p < 0.01, ***p < 0.001.

## Results

### Y110A mutant HALT-1 abrogates cytotoxic effects of wild-type HALT-1 on various cell lines

**Figure 1:**
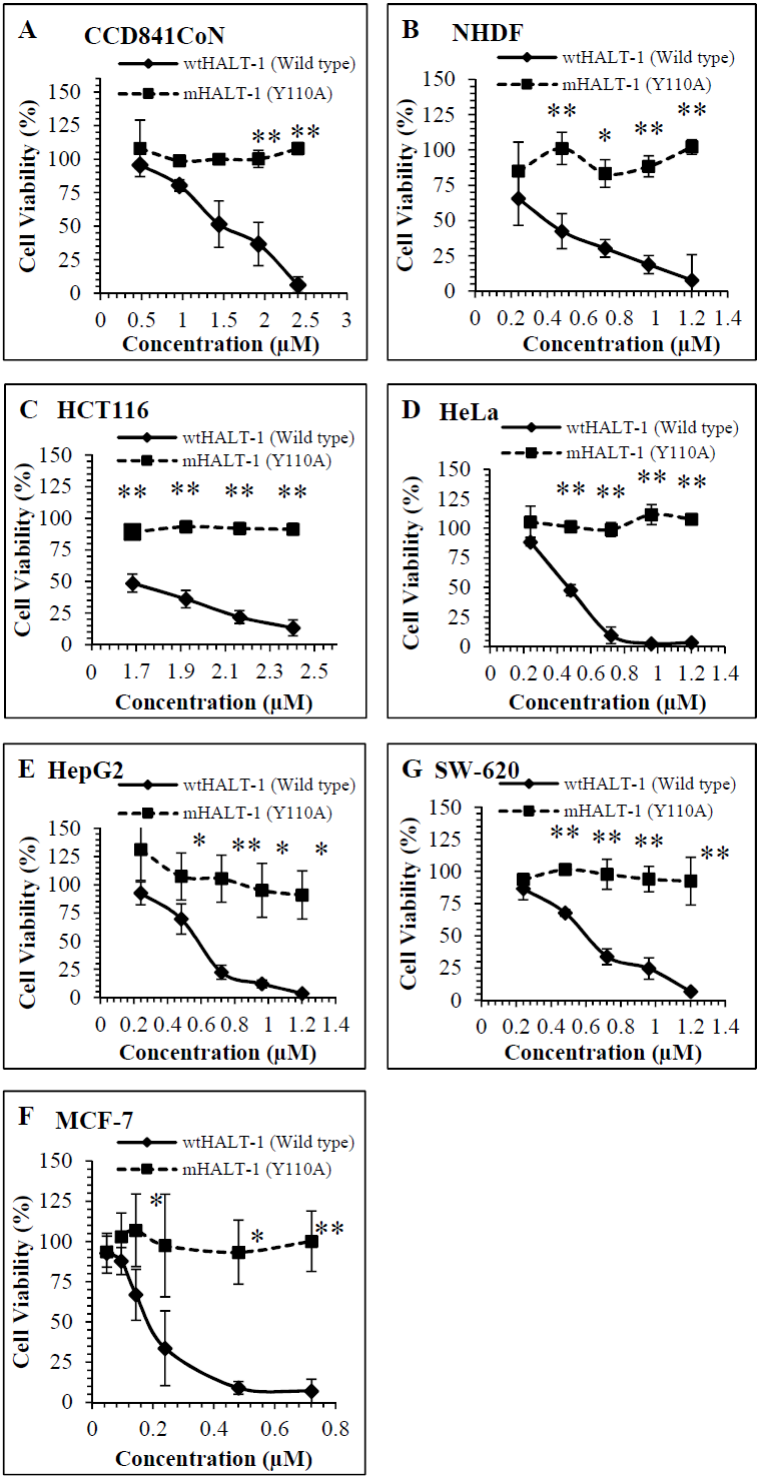
Comparison of cell viability on (A) CCD841CoN(B) NHDF (C) HCT116 (D)HeLa (E) HepG2 (F)MCF7 (G) SW-620 treated for 24 h with wtHALT-1 and mHALT-1. Data are represented as means  ± SD of three samples from three independent experiments, where (*) indicates significant difference levels as *p < 0.05, and **p < 0.01.

Dose dependent cytotoxic responses of wtHALT-1 and mHALT-1 on 5 cancer and 2 non-tumour cell lines are shown in Fig. 1. Experimental results indicated that wtHALT-1 induced potent cytotoxic effects (IC_50_ range of 0.30–1.62 µM) against human cancer cell lines, including MCF-7, HeLa, HepG2, HCT116 and SW-620 (Table 1). The IC_50_ of wtHALT-1 (0.51 µM) on HeLa cells was consistent with values obtained previously by (Liew et al., 2015) where HeLa cell viability dropped from 88% to 3% after wtHALT-1 treatment. Among all the cell lines tested, the potency of wtHALT-1 was found to be highest on MCF-7 cells with an IC_50_ value of 0.30 µM. The cytotoxic effect of wtHALT-1 was shown to be 50% stronger than mHALT-1 (*p* = 0.022) on MCF-7 cells (IC_50_ = 0.30 µM). Similar effects were observed on HepG2 (wtHALT-1 IC_50_ = 0.60 µM), where a significant reduction of mHALT-1 cytotoxicity (*p* = 0.004) was observed. The non-tumour human cell line (NHDF) also exhibited the same activity as HepG2 where viability levels were significantly higher (*p* = 0.008) at 92% after being treated with mHALT-1 in comparison to 33% with wtHALT-1 (IC_50_ of 0.52 µM). It was hypothesized that IC_50_ variations among cell lines were attributed to differences in SM distributions in the plasma membrane, which remains poorly studied to date (Makino et al., 2015). It was also interesting to note that IC_50_ values were higher in colorectal cell lines SW-620 (IC_50_ = 0.64 µM), HCT116 (IC_50_= 1.47 µM) and CCD841CoN (IC_50_ = 1.62 µM) in comparison to other cancer cell lines. At concentrations of 0.24, 0.48, 0.72, 0.96 and 1.20 µM of wtHALT-1, the cell viability of SW-620 were reported to be 86%, 68%, 34%, 25% and 7% respectively. Cell viability of SW-620 after treatment with mHALT-1 was significantly higher than its wild type counterpart (*p* = 0.021) with viability levels above 96%. The cytotoxicity pattern was similar on HCT116 and CCD841CoN where the viability of mHALT-1 was 2 to 3 times higher than wtHALT-1 (*p* = 0.005 and 0.039) respectively. Collectively, these results supported the notion that the Y110A mutation at the SM binding domain of mHALT-1 was successful in abrogating the cytotoxic effects of wtHALT-1.

**Table 1:** A summary of IC_50_ values for various cell lines (1 × 10^4^ cells/mL) treated for 24 h with wtHALT-1 and mHALT-1.

**Cell lines**	**Compound**	**IC**_**50**_	***R***^2^**value**
HeLa	wtHALT-1	0.51 µM	0.999
	mHALT-1	n/a	0.722
HepG2	wtHALT-1	0.60 µM	0.996
	mHALT-1	n/a	0.461
MCF-7	wtHALT-1	0.30 µM	0.999
	mHALT-1	n/a	0.393
SW-620	wtHALT-1	0.64 µM	0.986
	mHALT-1	n/a	0.777
HCT116	wtHALT-1	1.47 µM	0.969
	mHALT-1	n/a	0.576
NHDF	wtHALT-1	0.52 µM	0.994
	mHALT-1	n/a	0.323
CCD841CoN	wtHALT-1	1.62 µM	0.997
	mHALT-1	n/a	0.820

### wtHALT-1 induces apoptosis-mediated cell death

**Figure 2:**
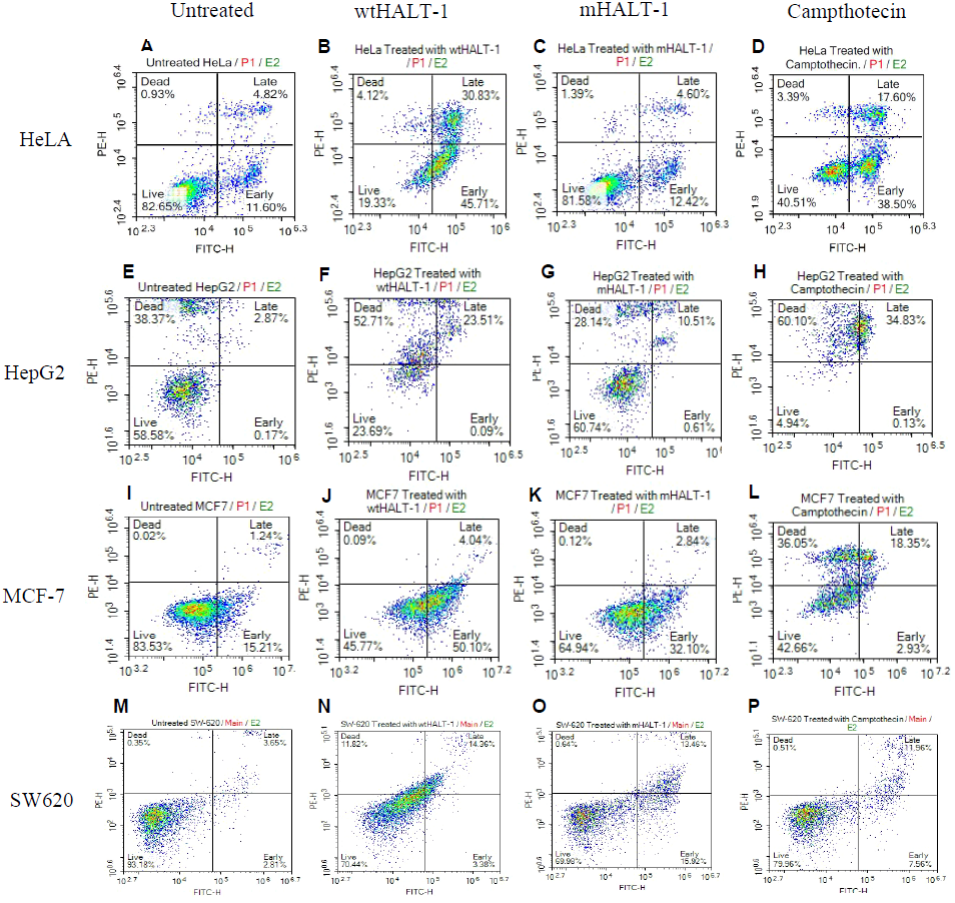
Annexin V/FITC-PI flow cytometry analysis of various cancer cells (1 × 10^6^ cells/mL) treated with negative control, wtHALT-1,mHALT-1 and camptothecin for 24 h. Cells were dual stained with Annexin V-FITC and propidium iodide, and the dot plot of HeLa, HepG2, MCF-7 and SW-620 with different treatments were shown. Each set of data shown are a representative plot of three independent experiments, while percentages are the mean value of three independent experiments.

Once HALT-1 cytotoxic properties were assessed, whether cell death was mediated by apoptosis was determined. Flow cytometry analysis with Annexin V-FITC/PI double staining was conducted where viable cells would remain unstained (Annexin V-FITC^−^/PI^−^), early apoptotic cells would be Annexin V-FITC^+^/PI^−^, whereas late apoptotic cells would exhibit a double positive Annexin V-FITC^+^/PI^+^ staining pattern due to loss of plasma membrane integrity and externalization of phosphatidylserine (Wlodkowic et al., 2011; Martin et al., 1995; Walsh et al., 1998; Brauchle et al., 2014). Exposure to wtHALT-1 at IC_50_ on HeLa cells for 24 h demonstrated an increase in early and late apoptotic cells at 35.50% and 22.81%, respectively (Fig. 2). In comparison, treatment with mHALT-1 yielded only 9.86% and 7.87% in early and late apoptosis (*p* = 0.164), which was consistent with preliminary MTT data. Camptothecin treated cells also demonstrated an increase in early and late apoptosis (35.96% and 19.74% respectively). Similar patterns of apoptotic induction between wtHALT-1 and mHALT-1 were also observed on MCF-7 cells (Fig. 2). These results confirmed that wtHALT-1 triggered cell death via an apoptosis-induced mechanism, albeit the type of apoptotic pathways involved remains unknown at present. However, the observed effects of wtHALT-1 on HepG2 were skewed more towards the necrotic population, presumably due to delayed post-treatment assay timing. As shown in Fig. 2, we noticed around 40% of untreated HepG2 had necrotic cells. It was also noteworthy that apoptotic effects were found to be inconsistent on SW-620 cells where mHALT-1 showed 7.12% higher levels of apoptotic cells than wtHALT-1 even after the membrane binding affinity was removed, possibly indicating that mHALT-1 is capable of binding to other membrane markers unique to this cell line with relatively low affinity in addition to sphingomyelin-based recognition.

### Loss of wtHALT-1 membrane bound SM affinity on various cell lines following Y110A mutation

**Figure 3:**
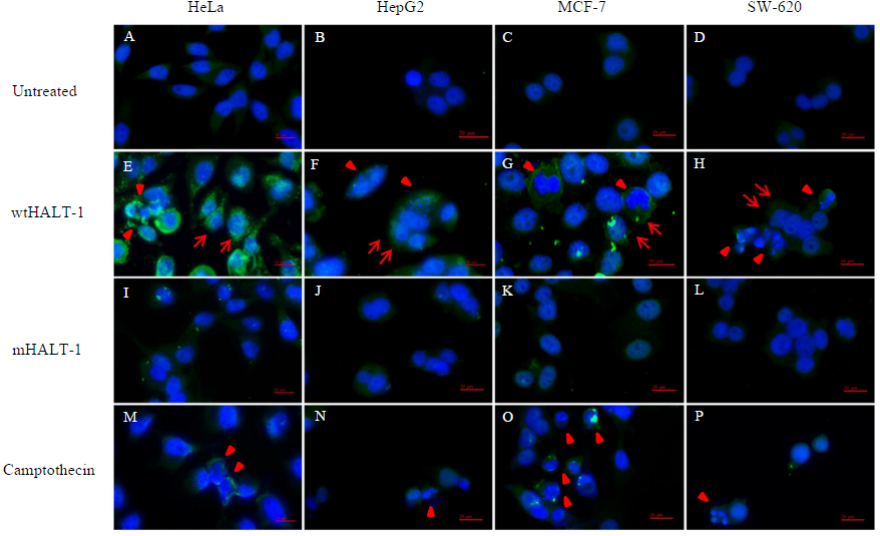
Immunofluoroscence images of cell lines treated with wtHALT-1, mHALT-1 and camptothecin for 24 h. Cells were stained with anti- His tag antibody (1:200) and stained immunofluoroscently with Alexa Fluor 488 goat anti-mouse IgG (H+L) antibody (1:3,000 dilutions) (green). Nuclei were counterstained with Hoechst dye (blue). Images were obtained on an Axio Vert A1 under 630×  magnification and arranged with Zen software, Adobe photoshop CC editor, and FotoJet. Red arrows indicate HALT-1 binding while arrow head indicate apoptotic cells. Scale bars: 20 µM. Each set of data shown are representative profile of three independent experiments.

**Figure 4:**
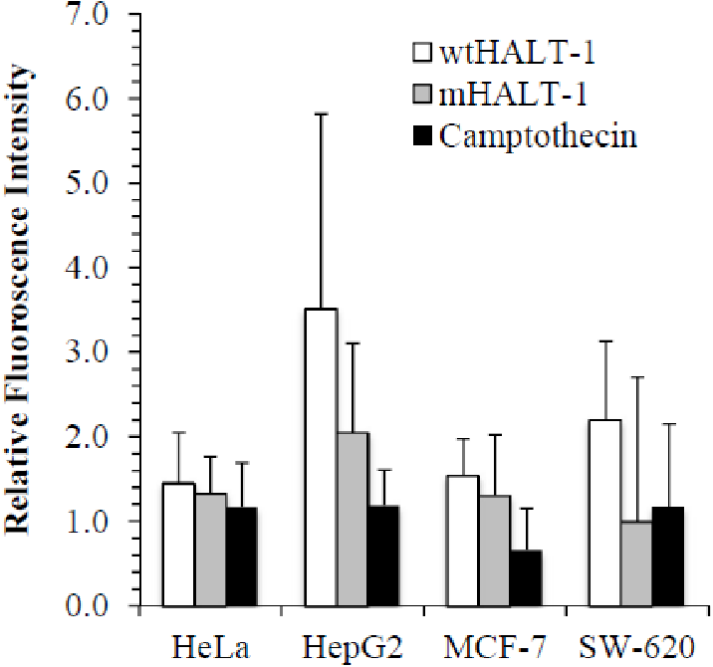
Quantification of immunofluorescent staining. HeLa, HepG2, MCF7 and SW-620 cell lines were seeded at 1 × 10^4^ cells/well in 96-well plates. Cell lines were treated with wtHALT-1, mHALT-1 and camptothecin at IC_50_ values. Fluorescence signal intensities were quantified with the Zen software and presented as mean values from triplicate experiments.

In order to study the localization of wtHALT-1 and mHALT-1, immunofluorescence staining was performed on HeLa, HepG2, MCF-7 and SW-620 cells. Different cell lines were incubated with different IC_50_ of wtHALT-1, mHALT-1 and camptothecin for 24 h followed by labeling with anti-His tag antibodies and stained fluorescently with Alexa Fluor 488-conjugated goat anti-mouse IgG (H+L) antibody. A significant difference between wtHALT-1 and mHALT-1 binding treatment was clearly observed where emission of green fluorescence along the cell membrane was observed only in the former (Fig. 3). Additionally, the occurrence of apoptosis on various cell lines after wtHALT-1 treatment and camptothecin treatment was also evident through cell morphological changes and nuclear condensation (Fig. 3). Relative fluorescence intensity for each cell line was normalized with the negative untreated control as depicted in Fig. 4. Relative fluorescence intensity upon treatment with mHALT-1 showed 0.12 fold reduction in comparison to wtHALT-1 treated HeLa cells. The biggest reduction of relative fluorescence intensity was observed on HepG2 cells, with a 41.6% reduction in mHALT-1 compared to wtHALT-1 treatment. Fluorescence intensity patterns in MCF-7 and SW-620 were also consistent between HeLa and HepG2 cells where a general reduction was observed between wtHALT-1 and mHALT-1, thus reaffirming the success of Y110A mutants in neutralizing binding capabilities of HALT-1.

## Discussion

In this study, wtHALT-1 was expected to bind to almost all cell lines tested, while a point mutation in wtHALT-1 at Y110A position was hypothesized to disable the toxin’s recognition capabilities towards human cell membranes. This is critical because as an immunotoxin candidate it should only bind specially to the targeted cells when coupled with an antibody or an equivalent targeting moiety. Actinoporins including HALT-1 are conserved in their structure where beta-sheets are sandwiched by two alpha-helices. Crystallographic study of fragaceatoxin C (sea anemone actinoporin) demonstrated van der Waals and nonpolar interactions between valine 60 (of one protomer) and phenylalanine 163 (of another protomer), and this bonding contributes to dimerization or oligomerization (Mesa-Galloso et al., 2016). These two residues are positioned distantly from tyrosine 110 in the 3D crystal structure, suggesting that tyrosine 110 does not play a role in oligomerization. Moreover, substitution of tyrosine 110 with alanine removes the benzene ring instead of introducing bulky groups, which may affect the protein structure. In a recent study, tyrosine at position 110 of HALT-1 was predicted to be crucial for sphingomyelin recognition and binding (Liew et al., 2015). Site directed mutagenesis removed the aromatic ring of the tyrosine amino acid essential for membrane recognition, and was performed previously producing the mHALT-1 variant used in this study (Bakra & Anderluh, 2010). Assessment on 7 cell lines in this study has demonstrated that wtHALT-1 significantly reduces cell viability at a lower IC_50_ compared to mHALT-1. Results in Fig. 1 showed that more than 50% of cells were non-viable after 24 h of treatment, comparable to Glasser et al. (2014) data, thus confirming the cytotoxic properties of wtHALT-1. This study indicates that mutants harbouring a single substitution from tyrosine to alanine reduces the cytotoxic activity of wtHALT-1 consistent with the result of Liew et al. (2015) who reported that HALT-1 reduced the viability of HeLa cells by 50% at 15 µg/mL but was maintained at more than 80% when treated with mHALT-1. It was hypothesized that the loss of its membrane binding ability was responsible for its inability to permeabilize the cell membrane to create pores.

Although wtHALT-1 showed cytotoxic effects on different cancer cell lines, it was not clear how this toxin induced cell death once introduced to cancer cell lines. In this study, results obtained from Annexin V FITC/PI apoptosis assay on four out of the five cancerous cell lines with low IC_50_ values revealed that cytotoxicity was triggered via an apoptosis-mediated cell death mechanism. These results are further supported by Jouiaei et al. (2015) which reported ß-PFT toxins derived from sea anemone *Phyllodiscus semoni* and *Actinaria villosa* were also capable of initiating apoptotic cell death pathways. Even though the annexin V-FITC/PI staining method has been well established, this method still suffers from several limitations in accurately measuring cells that have died of apoptosis versus necrosis. An example of this limitation is when the end point measurement is conducted exceeding the apoptotic window period, as apoptotic cells also lose membrane permeability in the later stages of apoptosis due to severe membrane damage, as was observed with HepG2 cells. Another noteworthy limitation with this assay when investigating pore forming toxins such as HALT-1 is the enhanced influx of PI into cells when pores are formed following wtHALT-1 binding, which can compromise the quantification of cell death between treatment groups.

While numerous experiments have supported the critical role for SM as the membrane attachment site for sea anemone actinoporins (Bakra & Anderluh, 2010) some reports suggest otherwise. A similar result was obtained with another actinoporin sticholysin of *Stichodactyla helianthus,* which was able to permeabilize large unilamellar vesicles (LUVs) composed of cholesterol and phosphatidylcholine in addition to SM (De los Ríos et al., 1998; Anderluh & Maček, 2003; Kristan et al., 2009). Another recent study also reported the folate receptor alpha as an interaction partner of HALT-1, which served as an alternative to the previously established binding of HALT-1 to the lipid SM (Ameirika, Sha & Hwang, 2017). Therefore, further developmental efforts may be required to ascertain whether optimization through additional point mutations at other binding domains of mHALT-1 are required to completely neutralize its binding properties, hence transitioning it into a promising toxin moiety candidate.

## Conclusions

In conclusion, the results of this investigation confirm the potential for mHALT-1 with a Y110A point substitution mutation as a potential toxin moiety candidate capable of inducing apoptosis for the development of future immunotoxins as a form of targeted therapy.

## Abbreviations

*α*-PFT: alpha—pore forming toxin
CEA: carcinoembryonic antigen
DMEM: Dulbecco’s minimal essential medium
EDTA: ethylenediamine tetraacetic acid
EqtII: equaitanoxin II
FBS: fetal bovine serum
HALT-1: *Hydra* actinoporin like toxin −1
IC_50_: inhibitory concentration
IF: immunofluorescence
IgG: immunoglobulin G
IPTG: isopropyl-beta-D-thiogalactopyranoside
MTT: 3-(4,5-Dimethylthiazol-2-yl)-2,5-diphenyltetrazolium bromide
NHDF: normal human dermal fibroblast
OD: optical density
PBS: phosphate buffer saline
PI: propidium iodide
PMSF: phenylmethylsulfonyl fluoride
POC: phosphocholine
SDS- PAGE: sodium dodecyl sulfate polyacrylamide gel electrophoresis
SM: sphingomyelin
StII: sticholysin II

## Supplemental Information

Figure S1 Cell viability of (A) MCF-7 (B) HeLa treated for24, 48 and 72 h with camptothecin as positive control. Data are represented as means ±SD from three independent experiments.

Figure S2 Time course comparison of cell viability for (A)MCF-7 (B) HeLa treated for 48 and 72 h with wtHALT-1 and mHALT-1. Data are represented as means ± SD from three independent experiments, where (*) indicates significant difference levels as **p* < 0.05, and ***p* < 0.01.

Table S1 A summary of IC_50_ values for MCF-7and HeLa cell lines (1 × 10^4^ cells/mL) treated for 24, 48 and 72 h with camptothecin as positive control, wtHALT-1 and mHALT-1.

Supplemental Information 1 Absorbance reading for wtHALT-1, mHALT-1 and campthothecin on CCD841CoN cells Minus blank is average value of each concentration subtracted with average of blank. Standard deviation is standard error calculated from the three readings.

Supplemental Information 2 Absorbance reading for wtHALT-1, mHALT-1 and camptothecin on HepG2 cells Minus blank is average value of each concentration subtracted with average of blank. Standard deviation is standard error calculated from the three readings.

Supplemental Information 3 Absorbance reading for wtHALT-1, mHALT-1 and camptothecin on MCF-7 cells Minus blank is average value of each concentration subtracted with average of blank. Standard deviation is standard error calculated from the three readings.

Supplemental Information 4 Absorbance reading for wtHALT-1, mHALT-1 and camptothecin on HeLa cells Minus blank is average value of each concentration subtracted with average of blank. Standard deviation is standard error calculated from the three readings.

Supplemental Information 5 Absorbance reading for wtHALT-1, mHALT-1 and camptothecin on HCT116 cells Minus blank is average value of each concentration subtracted with average of blank. Standard deviation is standard error calculated from the three readings.

Supplemental Information 6 Absorbance reading for wtHALT-1, mHALT-1 and camptothecin on NHDF cells Minus blank is average value of each concentration subtracted with average of blank. Standard deviation is standard error calculated from the three readings.

Supplemental Information 7 Absorbance reading for wtHALT-1, mHALT-1 and camptothecin on SW-620 cells Minus blank is average value of each concentration subtracted with average of blank. Standard deviation is standard error calculated from the three readings.

Supplemental Information 8 Analysis of HeLa cells ( 1 × 10^6^ cells/mL) HeLa cells were treated with negative control (untreated HeLa), wtHALT-1, mHALT-1 and camptothecin for 24 h. Cells were stained with Annexin V-FITC and propidium iodide. Dot plot of HeLa with different treatments were shown as three independent experiments.

Supplemental Information 9 Analysis of HepG2 cells ( 1 × 10^6^ cells/mL) HepG2 cells treated with negative control (untreated HepG2), wtHALT-1, mHALT-1 and camptothecin for 24 h. Cells were stained with Annexin V-FITC and propidium iodide. Dot plot of HepG2 with different treatments from three independent experiments were shown.

Supplemental Information 10 Analysis of SW-620 cells ( 1 × 10^6^ cells/mL) SW-620 cells were treated with negative control (untreated SW-620), wtHALT-1, mHALT-1 and camptothecin for 24 h. Cells were stained with Annexin V-FITC and propidium iodide. Dot plot of SW-620 with different treatments from three independent experiments were shown.

Supplemental Information 11 Analysis of MCF-7 cells ( 1 × 10^6^ cells/mL) MCF-7 cells were treated with negative control (untreated MCF-7), wtHALT-1, mHALT-1 and camptothecin for 24 h. Cells were stained with Annexin V-FITC and propidium iodide. Dot plot of MCF-7 with different treatments from three independent experiments were shown.

Supplemental Information 12 Percentage of apoptosis-inducing effects on various cancer cell lines *p*-values presented were obtained using two-tailed *t*-test by comparing wtHALT-1 and mHALT-1 on various cell lines. Data significance levels were shown as * *p* < 0.05, ** *p* < 0.01, *** *p* < 0.001.

Supplemental Information 13 Immunofluorescence images of HeLa cells. <!–[if !mso]–><!–[endif]–><!–[if !mso]–><!–[endif]–><!–[if !mso]–><!–[endif]–><!–[if !mso]–><!–[endif]–><!–[if !mso]–>< HeLa cells were treated with wtHALT-1, mHALT-1 and camptothecin for 24 h. Cells were stained with anti- His tag antibody (1:200) and stained immunofluorescently with Alexa Fluor 488 goat anti-mouse IgG (H+L) secondary antibody (1:3,000 dilutions) (green). Nuclei were counterstained with Hoechst dye (blue). Images were obtained on an Axio Vert A1 under 630×magnification and arranged with Zen software, Adobe photoshop CC editor, and FotoJet. White arrows indicate HALT-1 binding while arrow head indicate apoptotic cells. Scale bars: 20 µm. Each set of data shown are representative profile of three independent experiments.

Supplemental Information 14 Immunofluorescence images of HepG2 cells HepG2 cells were treated with wtHALT-1, mHALT-1 and camptothecin for 24 h. Cells were stained with anti- His tag antibody (1:200) and stained immunofluorescently with Alexa Fluor 488 goat anti-mouse IgG (H+L) antibody (1:3,000 dilutions) (green). Nuclei were counterstained with Hoechst dye (blue). Images were obtained on an Axio Vert A1 under 630×magnification and arranged with Zen software, Adobe photoshop CC editor, and FotoJet. White arrows indicate HALT-1 binding while arrow head indicate apoptotic cells. Scale bars: 20 µm. Each set of data shown are representative profile of three independent experiments.

Supplemental Information 15 Immunofluorescence images of MCF-7 cells MCF-7 cells were treated with wtHALT-1, mHALT-1 and camptothecin for 24 h. Cells were stained with anti- His tag antibody (1:200) and stained immunofluorescently with Alexa Fluor 488 goat anti-mouse IgG (H+L) antibody (1:3,000 dilutions) (green). Nuclei were counterstained with Hoechst dye (blue). Images were obtained on an Axio Vert A1 under 630×magnification and arranged with Zen software, Adobe photoshop CC editor, and FotoJet. White arrows indicate HALT-1 binding while arrow head indicate apoptotic cells. Scale bars: 20 µm. Each set of data shown are representative profile of three independent experiments.

Supplemental Information 16 Immunofluoroscence images of SW-620 cells SW-620 cells were treated with wtHALT-1, mHALT-1 and camptothecin for 24 h. Cells were stained with anti- His tag antibody (1:200) and stained immunofluoroscently with Alexa Fluor 488 goat anti-mouse IgG (H+L) antibody (1:3,000 dilutions) (green). Nuclei were counterstained with Hoechst dye (blue). Images were obtained on an Axio Vert A1 under 630×magnification and arranged with Zen software, Adobe photoshop CC editor, and FotoJet. Red arrows indicate HALT-1 binding while arrow head indicate apoptotic cells. Scale bars: 20 µm. Each set of data shown are representative profile of three independent experiments.

Supplemental Information 17 Fluorescence intensity of various cancer cell lines *p*-values presented were obtained using two-tailed *t*-test by comparing wtHALT-1 and mHALT-1 on HeLa cells. Data significance levels were shown as * *p* < 0.05, ** *p* < 0.01, *** *p* < 0.001.

### Additional Information and Declarations

#### Data Availability

The following information was supplied regarding data availability: The raw measurements and data are available in the Supplemental Files. The raw data includes MTT data, IF data and flow cytometry data showing the potential for mHALT-1 with a point substitution mutation as a potential toxin moiety candidate capable of inducing apoptosis for the developmental of future immunotoxins as a form of targeted therapy.
