# Supplementary figures and images for: Cytotoxic and apoptosis-inducing effects of wildtype and mutated Hydra actinoporin-like toxin 1 (HALT-1) on various cancer cell lines
Source: PeerJ. 2019 May 2;7:e6639. doi: 10.7717/peerj.6639 (PMC6500716; doi:10.7717/peerj.6639)

Supplementary Material - Figure 1

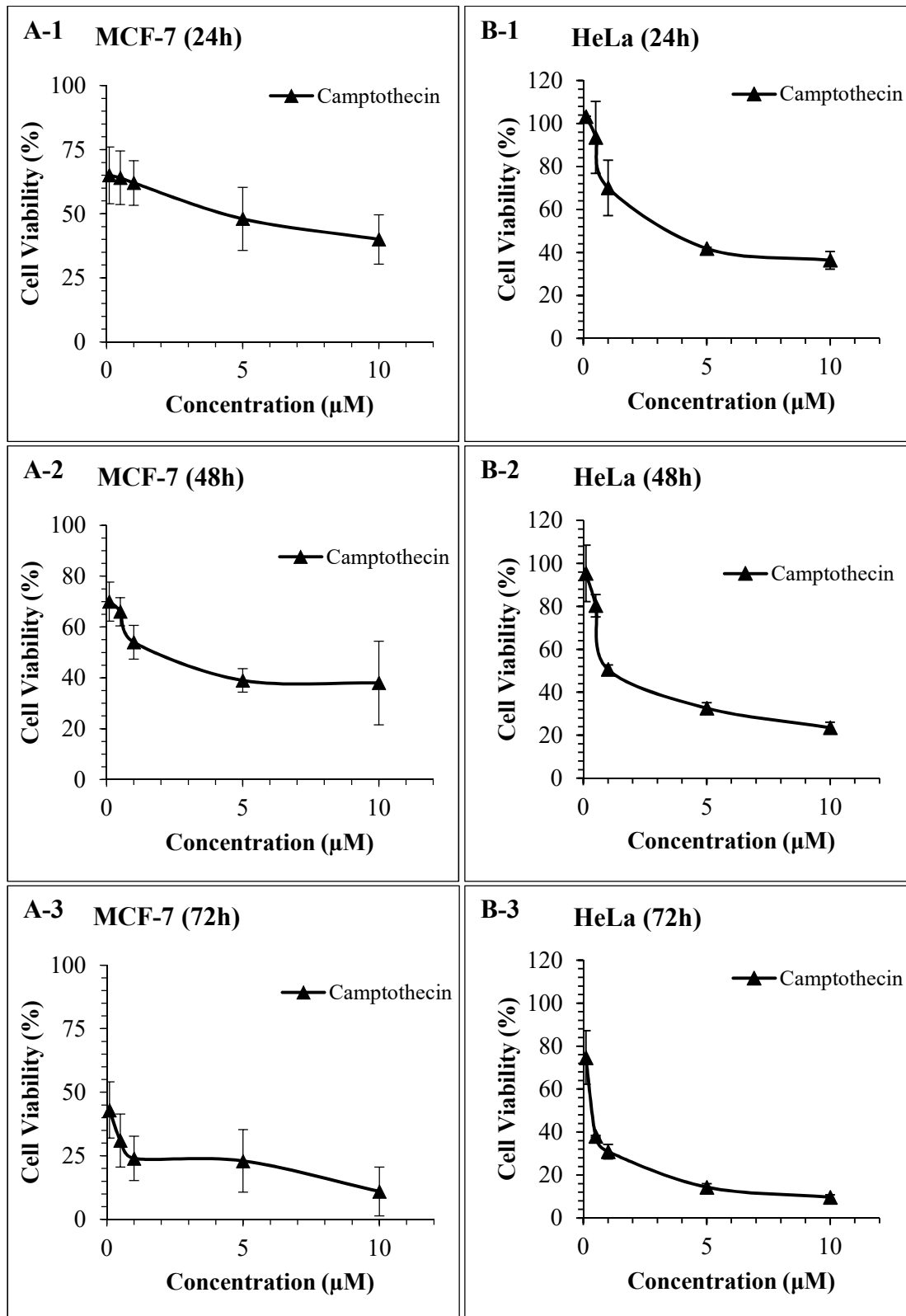

Supplement: Figure S1 — Data are represented as means ±SD from three independent experiments. [file peerj-07-6639-s001.pdf]

Supplementary Material - Figure 2

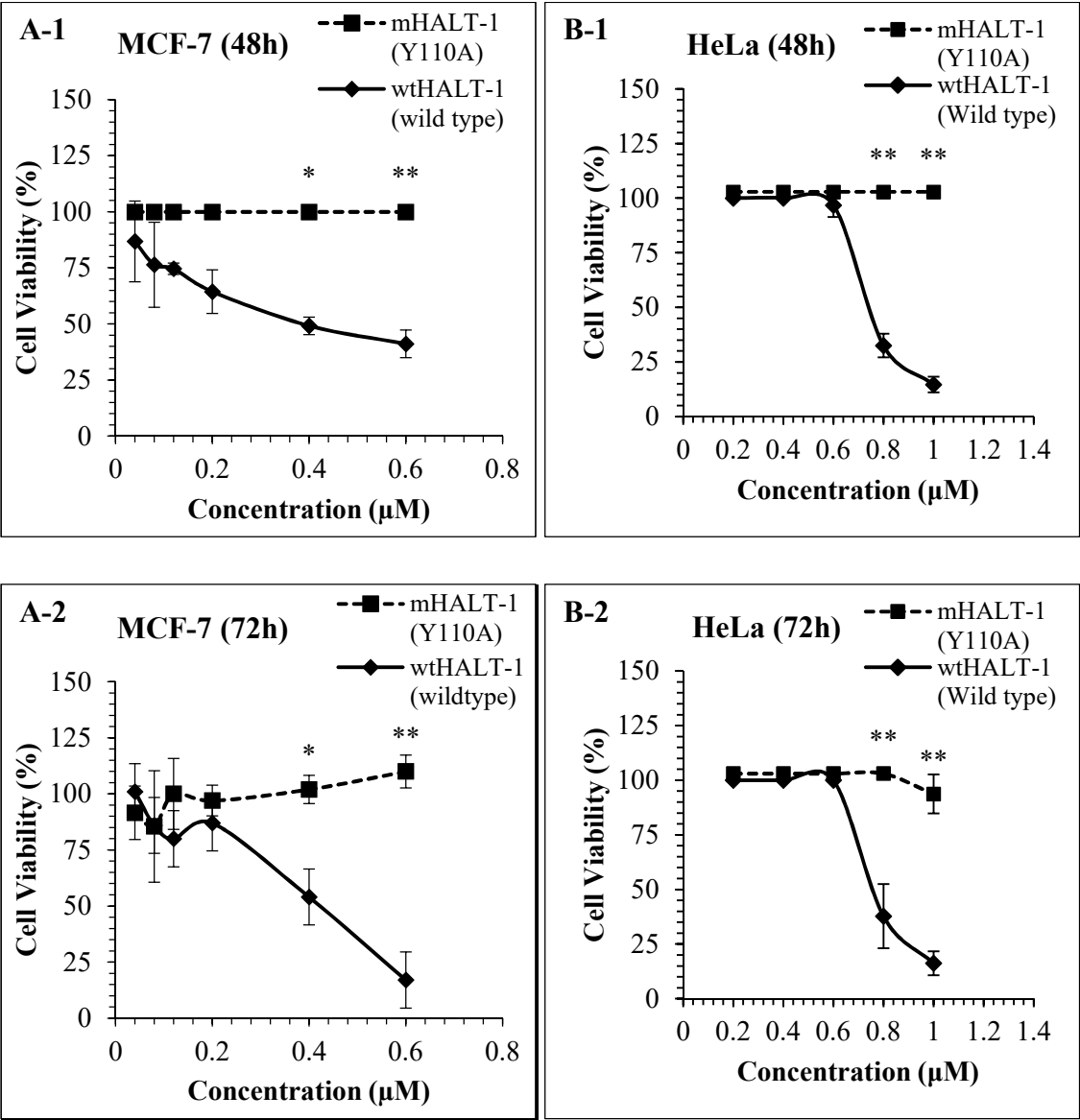

Supplement: Figure S2 — Data are represented as means ± SD from three independent experiments, where (*) indicates significant difference levels as *p < 0.05, and **p < 0.01. [file peerj-07-6639-s002.pdf]

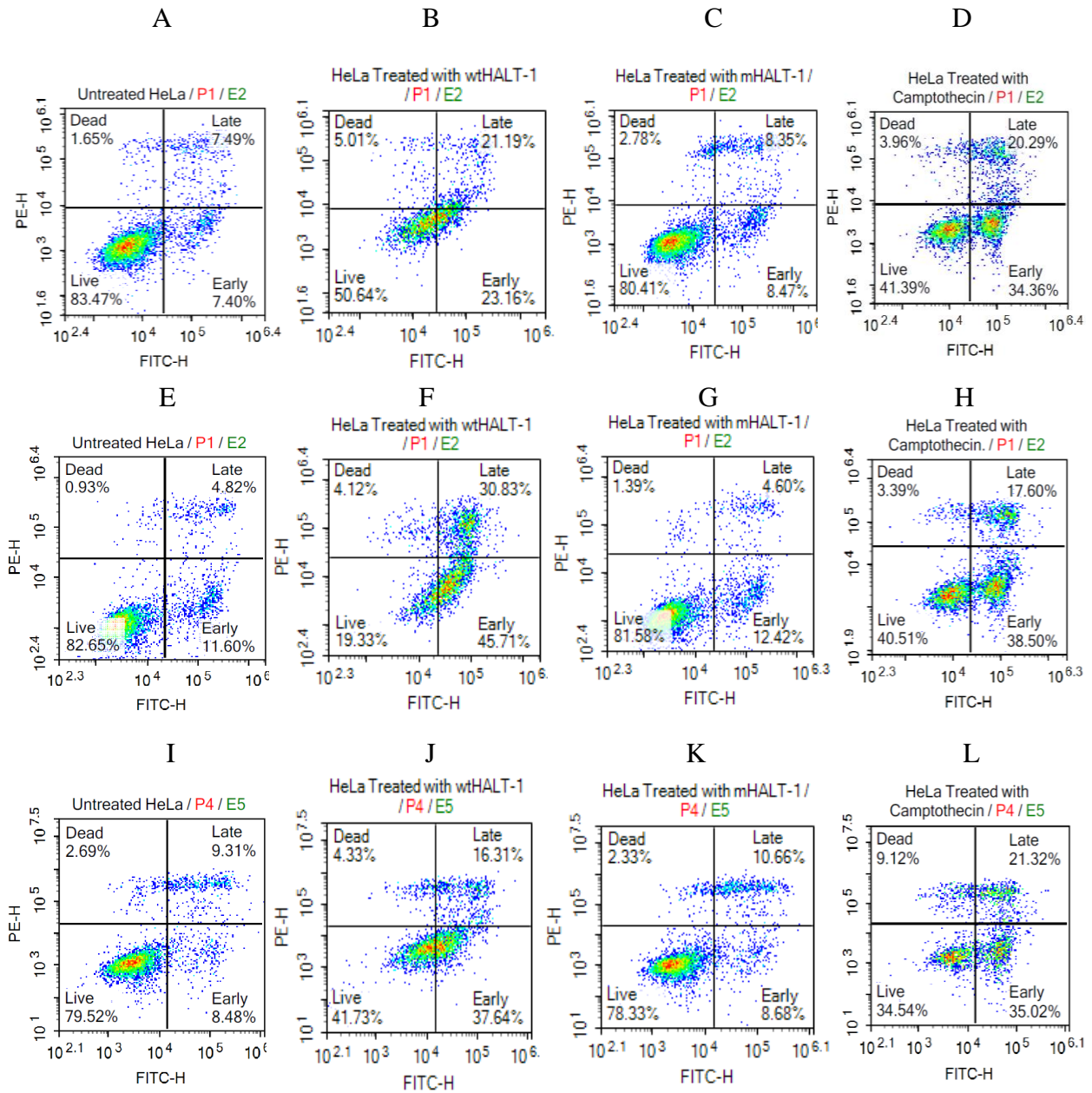

Supplement: Supplemental Information 8 — HeLa cells were treated with negative control (untreated HeLa), wtHALT-1, mHALT-1 and camptothecin for 24 h. Cells were stained with Annexin V-FITC and propidium iodide. Dot plot of HeLa with different treatments were shown as three independent experiments. [file peerj-07-6639-s011.pdf]

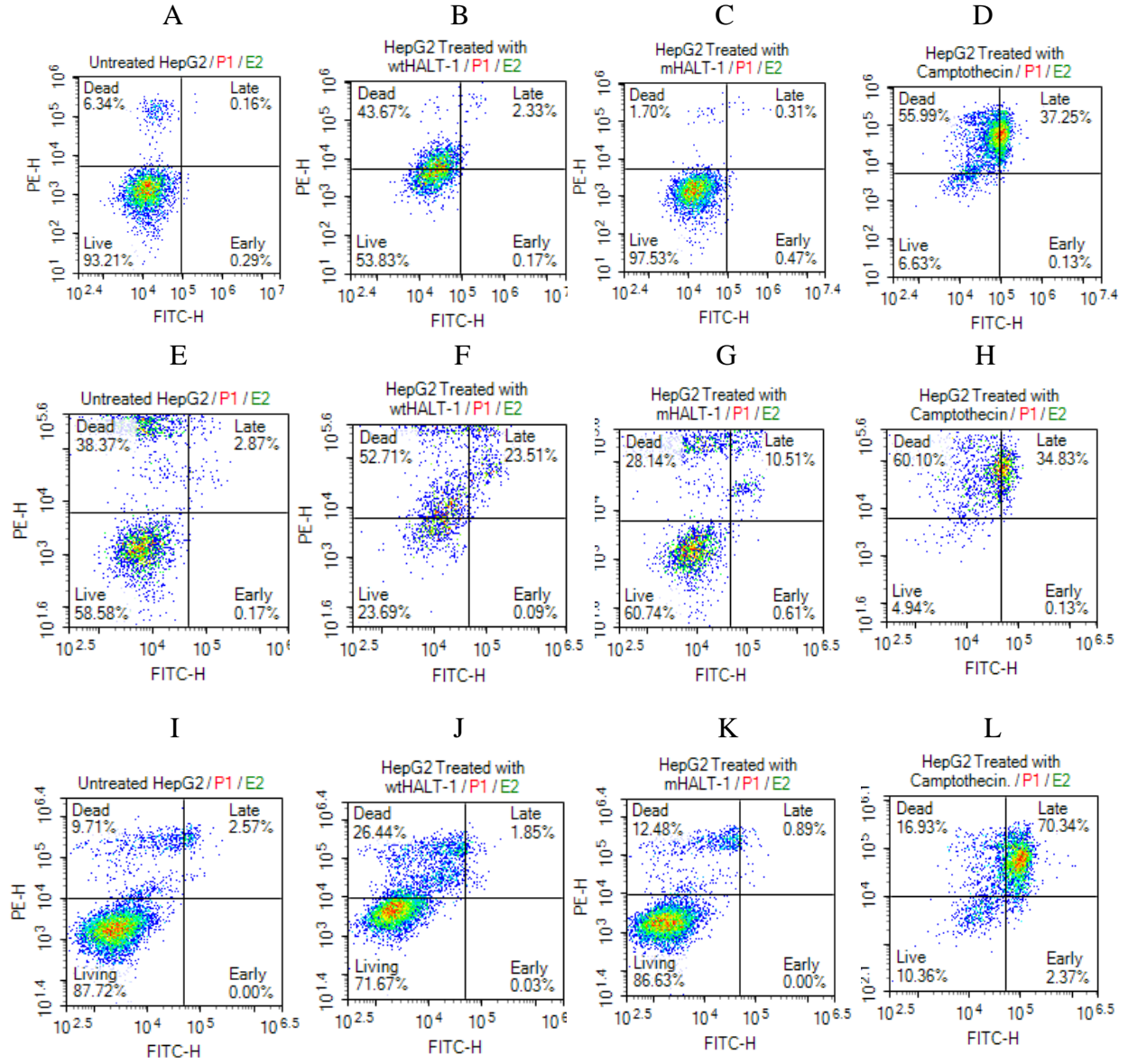

Supplement: Supplemental Information 9 — HepG2 cells treated with negative control (untreated HepG2), wtHALT-1, mHALT-1 and camptothecin for 24 h. Cells were stained with Annexin V-FITC and propidium iodide. Dot plot of HepG2 with different treatments from three independent experiments were shown. [file peerj-07-6639-s012.pdf]

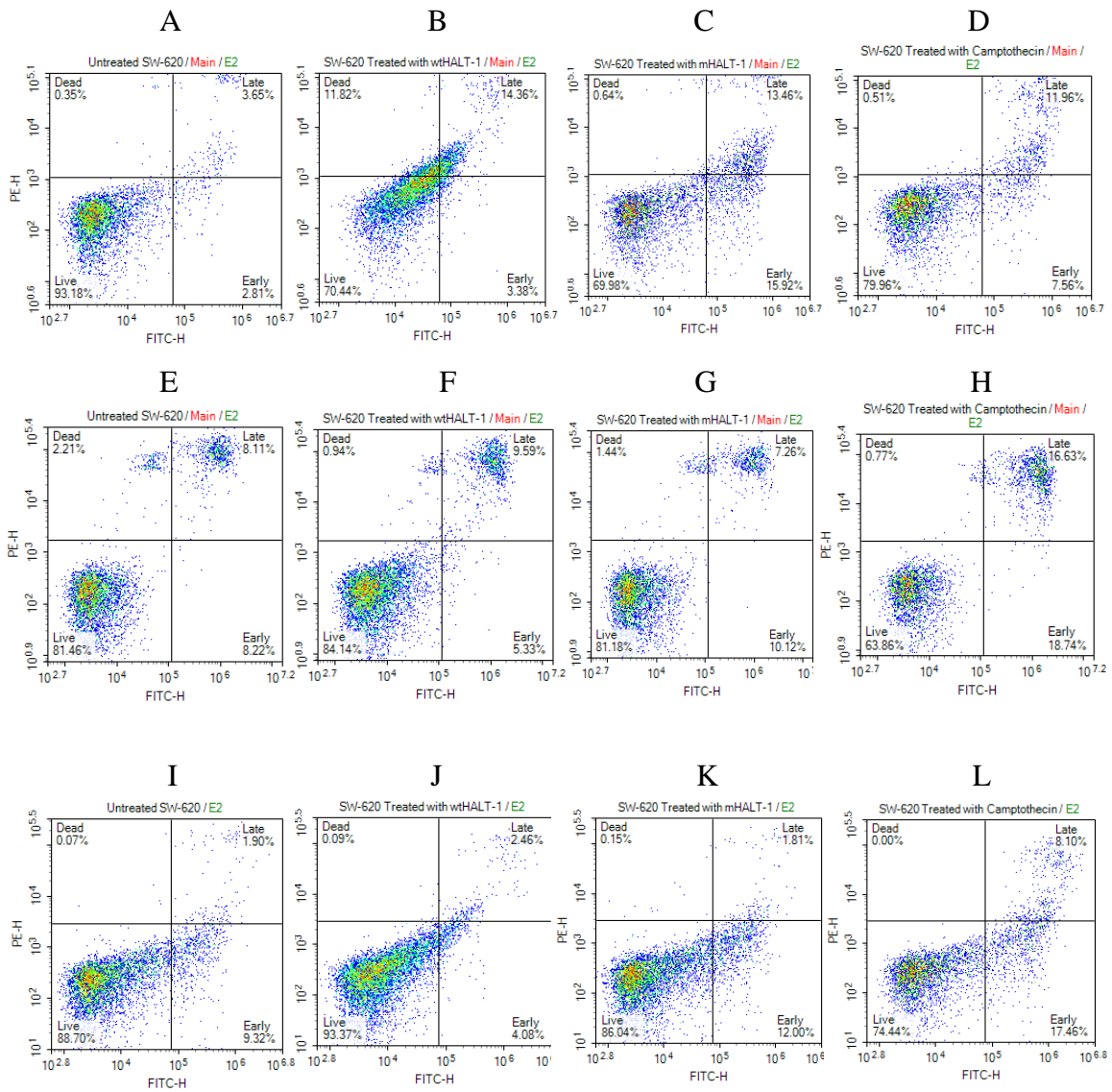

Supplement: Supplemental Information 10 — SW-620 cells were treated with negative control (untreated SW-620), wtHALT-1, mHALT-1 and camptothecin for 24 h. Cells were stained with Annexin V-FITC and propidium iodide. Dot plot of SW-620 with different treatments from three independent experiments were shown. [file peerj-07-6639-s013.pdf]

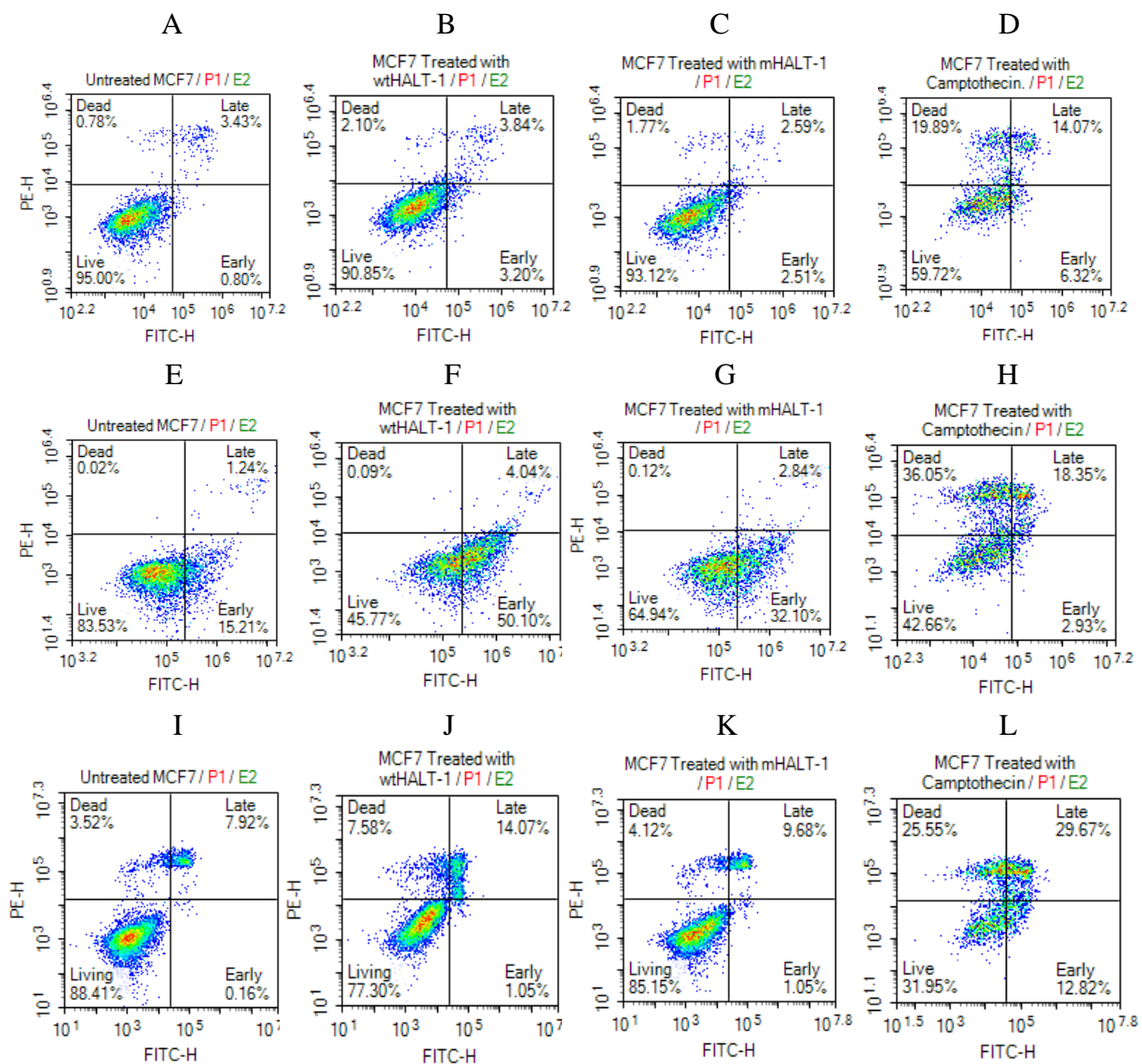

Supplement: Supplemental Information 11 — MCF-7 cells were treated with negative control (untreated MCF-7), wtHALT-1, mHALT-1 and camptothecin for 24 h. Cells were stained with Annexin V-FITC and propidium iodide. Dot plot of MCF-7 with different treatments from three independent experiments were shown. [file peerj-07-6639-s014.pdf]

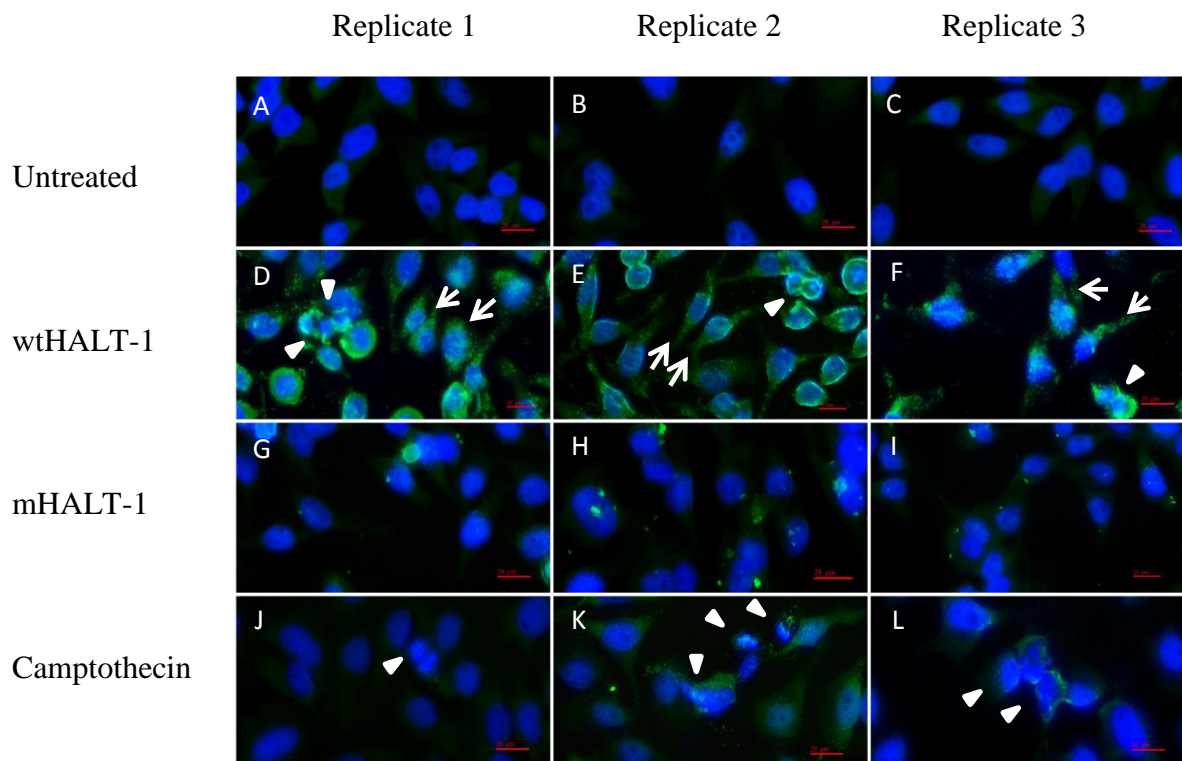

Supplement: Supplemental Information 13 — HeLa cells were treated with wtHALT-1, mHALT-1 and camptothecin for 24 h. Cells were stained with anti- His tag antibody (1:200) and stained immunofluorescently with Alexa Fluor 488 goat anti-mouse IgG (H+L) secondary antibody (1:3,000 dilutions) (green). Nuclei were counterstained with Hoechst dye (blue). Images were obtained on an Axio Vert A1 under 630×magnification and arranged with Zen software, Adobe photoshop CC editor, and FotoJet. White arrows indicate HALT-1 binding while arrow head indicate apoptotic cells. Scale bars: 20 µm. Each set of data shown are representative profile of three independent experiments. [file peerj-07-6639-s016.pdf]

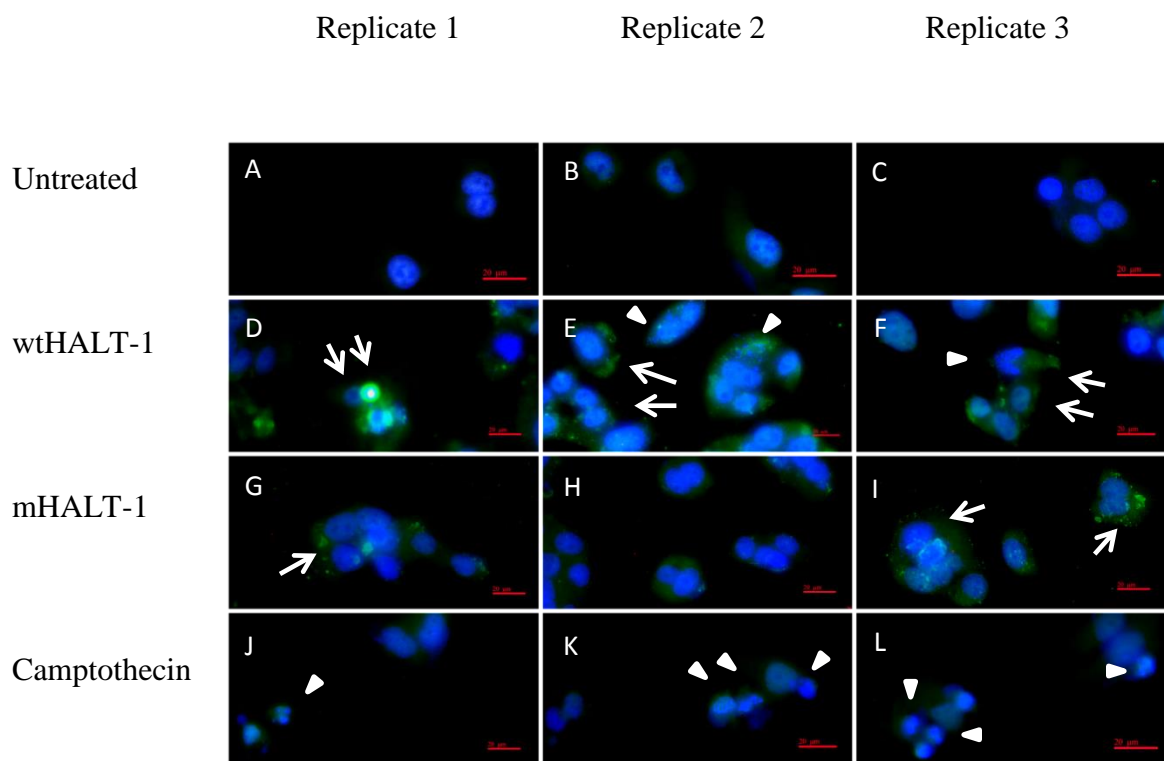

Supplement: Supplemental Information 14 — HepG2 cells were treated with wtHALT-1, mHALT-1 and camptothecin for 24 h. Cells were stained with anti- His tag antibody (1:200) and stained immunofluorescently with Alexa Fluor 488 goat anti-mouse IgG (H+L) antibody (1:3,000 dilutions) (green). Nuclei were counterstained with Hoechst dye (blue). Images were obtained on an Axio Vert A1 under 630×magnification and arranged with Zen software, Adobe photoshop CC editor, and FotoJet. White arrows indicate HALT-1 binding while arrow head indicate apoptotic cells. Scale bars: 20 µm. Each set of data shown are representative profile of three independent experiments. [file peerj-07-6639-s017.pdf]

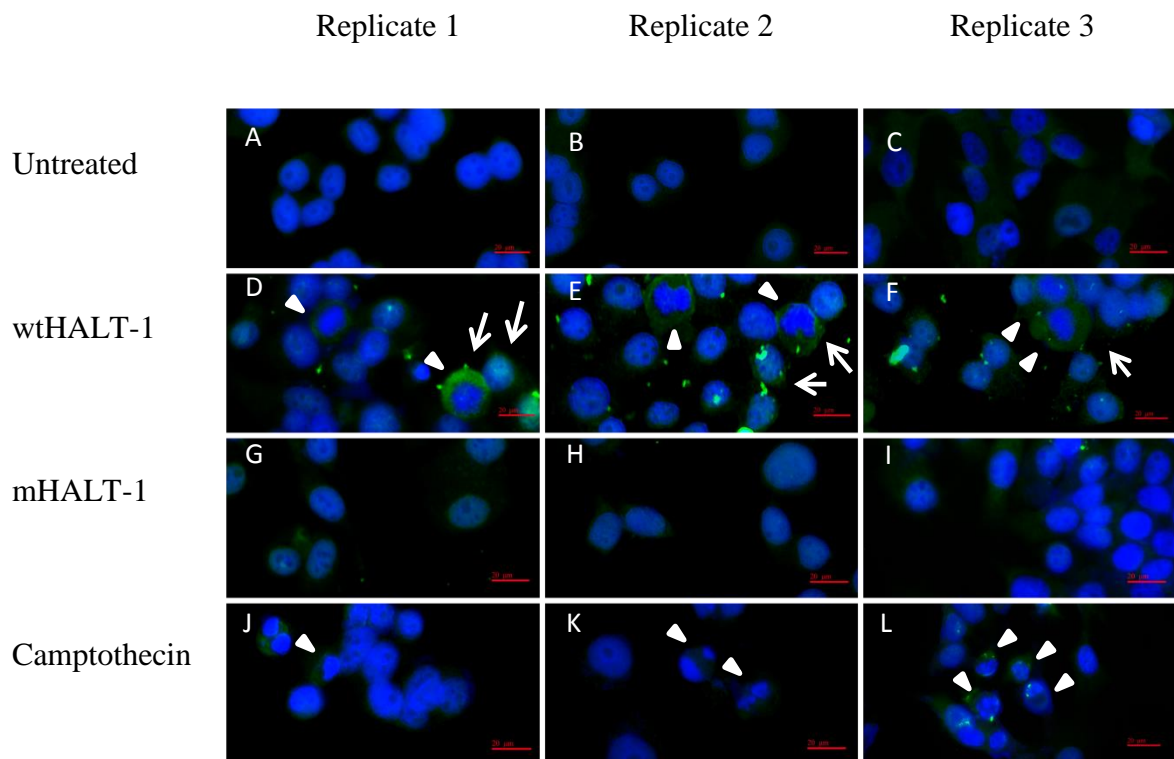

Supplement: Supplemental Information 15 — MCF-7 cells were treated with wtHALT-1, mHALT-1 and camptothecin for 24 h. Cells were stained with anti- His tag antibody (1:200) and stained immunofluorescently with Alexa Fluor 488 goat anti-mouse IgG (H+L) antibody (1:3,000 dilutions) (green). Nuclei were counterstained with Hoechst dye (blue). Images were obtained on an Axio Vert A1 under 630×magnification and arranged with Zen software, Adobe photoshop CC editor, and FotoJet. White arrows indicate HALT-1 binding while arrow head indicate apoptotic cells. Scale bars: 20 µm. Each set of data shown are representative profile of three independent experiments. [file peerj-07-6639-s018.pdf]

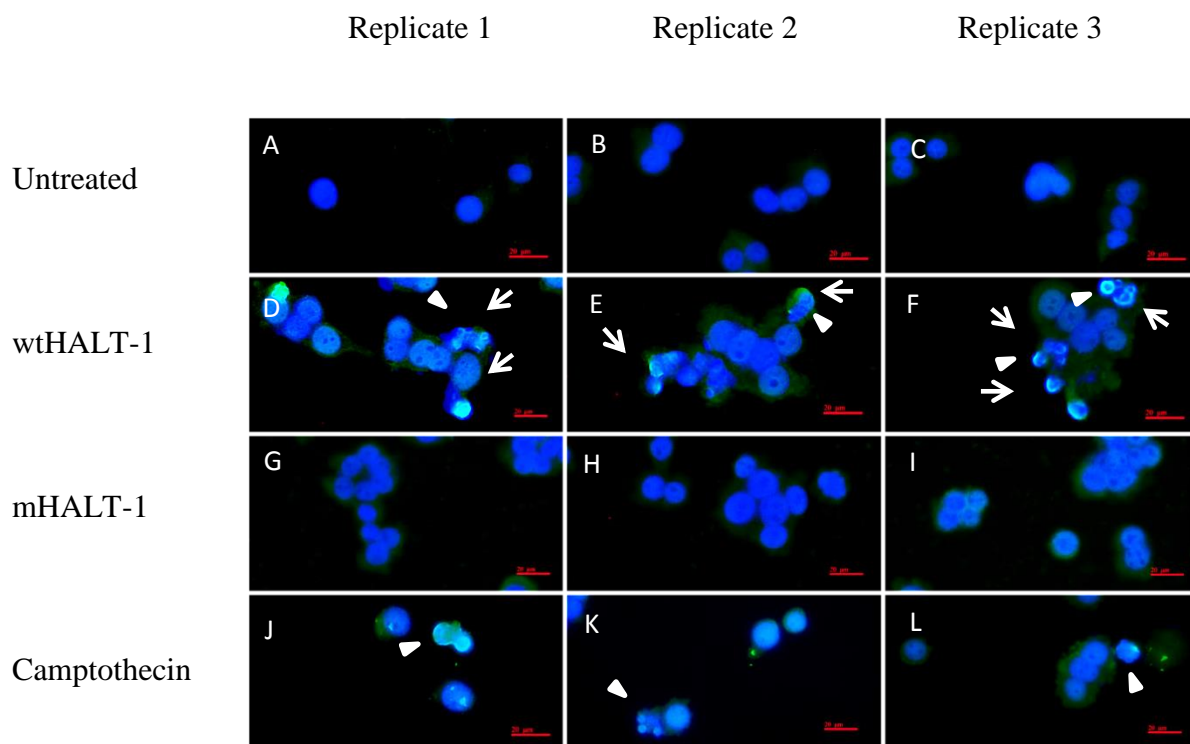

Supplement: Supplemental Information 16 — SW-620 cells were treated with wtHALT-1, mHALT-1 and camptothecin for 24 h. Cells were stained with anti- His tag antibody (1:200) and stained immunofluoroscently with Alexa Fluor 488 goat anti-mouse IgG (H+L) antibody (1:3,000 dilutions) (green). Nuclei were counterstained with Hoechst dye (blue). Images were obtained on an Axio Vert A1 under 630×magnification and arranged with Zen software, Adobe photoshop CC editor, and FotoJet. Red arrows indicate HALT-1 binding while arrow head indicate apoptotic cells. Scale bars: 20 µm. Each set of data shown are representative profile of three independent experiments. [file peerj-07-6639-s019.pdf]
