# Supplementary material for: Cytotoxic and apoptosis-inducing effects of wildtype and mutated Hydra actinoporin-like toxin 1 (HALT-1) on various cancer cell lines
Source: PeerJ. 2019 May 2;7:e6639. doi: 10.7717/peerj.6639 (PMC6500716; doi:10.7717/peerj.6639)
Supplement: Table S1 [file peerj-07-6639-s003.docx]

**Supplementary Material - Table 1**

| Cell lines | Time (h) | IC_50_ Camptothecin (µM) |
| --- | --- | --- |
| MCF-7 | 24 | 5.00 |
|  | 48 | 1.80 |
|  | 72 | 0.02 |
| HeLa | 24 | 4.35 |
|  | 48 | 2.57 |
|  | 72 | 0.05 |

**Supplementary Material - Table 2**

| Cell lines | Time (h) | IC_50_ wtHALT-1 (µM) | IC_50_ mtHALT-1 (µM) |
| --- | --- | --- | --- |
| MCF-7 | 48 | 0.42 | n/a |
|  | 72 | 0.39 | n/a |
| HeLa | 48 | 0.52 | n/a |
|  | 72 | 0.55 | n/a |
